# Uncertainties in the 2004 Sumatra–Andaman source through nonlinear stochastic inversion of tsunami waves

**DOI:** 10.1098/rspa.2017.0353

**Published:** 2017-09-20

**Authors:** D. Gopinathan, M. Venugopal, D. Roy, K. Rajendran, S. Guillas, F. Dias

**Affiliations:** 1 Department of Statistical Science, University College London, London WC1E 6BT, UK; 2 Department of Instrumentation and Applied Physics, Indian Institute of Science, Bangalore 560012, India; 3 Computational Mechanics Laboratory, Department of Civil Engineering, Indian Institute of Science, Bangalore 560012, India; 4 Centre for Earth Sciences, Indian Institute of Science, Bangalore 560012, India; 5 School of Mathematics and Statistics, University College Dublin, Dublin 4, Ireland

**Keywords:** tsunami simulation, earthquake source, inverse problem, uncertainty quantification

## Abstract

Numerical inversions for earthquake source parameters from tsunami wave data usually incorporate subjective elements to stabilize the search. In addition, noisy and possibly insufficient data result in instability and non-uniqueness in most deterministic inversions, which are barely acknowledged. Here, we employ the satellite altimetry data for the 2004 Sumatra–Andaman tsunami event to invert the source parameters. We also include kinematic parameters that improve the description of tsunami generation and propagation, especially near the source. Using a finite fault model that represents the extent of rupture and the geometry of the trench, we perform a new type of nonlinear joint inversion of the slips, rupture velocities and rise times with minimal *a priori* constraints. Despite persistently good waveform fits, large uncertainties in the joint parameter distribution constitute a remarkable feature of the inversion. These uncertainties suggest that objective inversion strategies should incorporate more sophisticated physical models of seabed deformation in order to significantly improve the performance of early warning systems.

## 1. Introduction

**Figure 1.**
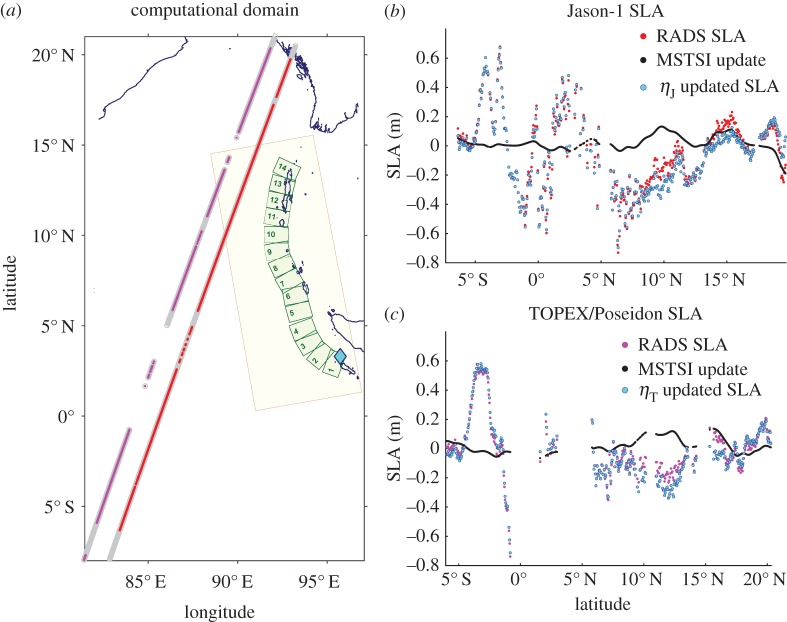
(*a*) The rupture is modelled by a 14-segment finite fault (FF) model (*green* boxes). The epicentre (*cyan* diamond) is located in the first segment. Satellite tracks (*grey*) with valid sea-level altimetry (SLA) locations for Jason-1 (*red*) and T/P (*magenta*). The near-field basin (*yellow box*) for uncertainty propagation. The measurement for the inversion is: (*b*) Jason-1 and (*c*) T/P SLA. The multi-satellite time-spatial interpolation (MSTSI) algorithm detects and removes anomalies (*black*) not pertaining to the tsunami, e.g. in the range 8°–13°*N*. Observe the near-parallel nature of the tracks to the rupture that enables the inversion of the dynamics for the entire rupture. No interpolation or addition of points is performed to give due importance to the available SLA data.

An unexpected opportunity for inversion of earthquake source parameters arose in the fortuitous detection of the tsunami caused by the 2004 Sumatra–Andaman event (2004 S-A) by satellites [1–3] (figure 1). The tsunami sea-level anomaly (SLA) detected by Jason-1 and TOPEX/Poseidon (T/P) altimeter-equipped satellites has no historical antecedent in its unambiguous demarcation of the signal and detection of its amplitude (approx. 0.6 m). Not only is the SLA capture duration commensurate with the entire rupture duration (approx. 600 s) but also the satellite track is near-parallel to the entire rupture length (approx. 1400 km). Given the rarity of tsunami detection by satellites, confluence of these remarkable features endows the SLA data with unprecedented value for inverting the source dynamics over the entire duration of the event. The corresponding uncertainty quantification affords a rare insight into the limitations in the underlying physical models of tsunami genesis. Moreover, the SLA data contain many embedded uncertainties [4]. The presence of these uncertainties further motivates the need for an inversion method that has the ability to quantify the consequent uncertainties in the inverted parameters.

Inversion of measured data for earthquake sources inevitably depends on the way the problem is posed and parametrized. Ill-conditioning and sensitivity issues may result from inaccurate physical models and inadequate or noisy measurements. Imposition of *a priori* subjective constraints (or regularization) is a common approach to the computational, if unphysical, amelioration of such drawbacks. Widely employed deterministic inversion schemes (e.g. the non-negative least-squares method) cannot, by nature, comprehensively handle measurement noise, parameter uncertainties and multiple possible solutions. These methods, nevertheless, provide insights into possible solutions to a nonlinear inversion, undertaken here for the slips, rupture velocities and rise times. They are not designed to quantify the uncertainties about the solutions in terms of the ranges of likely values resulting from the inversion, or the nature of the distributions over these ranges—both necessary to assess the validity of scientific deductions that ensue from the inversion. While probabilistic methods endeavour to amend such pitfalls of deterministic methods, many are ill-equipped for a global search, so a careful choice of the approach is a necessity when embarking on a probabilistic nonlinear inversion path. Finally, a single objective functional in the form of an error norm, employed in most of these schemes, may disregard local misfits and thus distort the statistical distribution of some of the inverted parameters whose influences might be purely local. There is thus a case for a stochastic and multi-objective global search *en route* to a more informative reconstruction. It may be noted here that despite their shortcomings, regularized versions of deterministic methods are paramount (and they remain the only available methods) in real-time tsunami warning systems where computational speed and response are critical.

The term ‘joint’ inversion usually denotes the inversion of multiple measurement types, e.g. tide gauge, SLA, geodetic, etc. in the geophysical community. However, in this work, the term ‘joint’ refers to the inversion for multiple parameter types, e.g. slips, rupture velocities and rise times while using only the SLA as the measurement. Lorito *et al.* [5] performed a nonlinear inversion for some of the kinematics rupture parameters (slip, rake and rupture velocity). However, this inversion did not include kinematic parameters that have been shown recently to be the key descriptors of the tsunami generation for the 2011 Japan Tohoku event [6]. Furthermore, Dutykh *et al.* [7] illustrated on the Java 2006 event the importance of the dynamics of the rupture process by constructing the dynamic seabed displacement according to the rupture propagation speed and the rise time. Lorito *et al.* [5] introduced steps of 1 m in the slip values on each subfault and 0.25 km s^−1^ steps in rupture velocity (and only employed four rupture velocities for the entire rupture, and did not vary rise times). This discretization of the search can only produce a partial understanding of the uncertainties. Although we chose one rupture velocity per segment (14 in total), we did not split the segments in the down-dip direction as Lorito *et al.* [5] did. Indeed, we argue that in order to reconstruct the satellite altimetry (near-parallel to the rupture), an increased resolution along the rupture is likely to be more informative for the reconstruction than along the down-dip direction.

Tanioka *et al.* [8] quantified uncertainties in slips using the non-negative least-squares method coupled with jackknifing, but assumed a constant rise time (120 s) for each segment; in addition, they selected, not jointly inverted, the rupture velocity from a discrete set of values. A similar procedure is performed by Hirata *et al.* [9] by assuming constant rise times for each segment (here 150 s). Satake *et al.* [10] assume five linear rise time functions (30 s each), which together, form a piecewise linear rise time function per segment. This can be seen as an indirect inversion of rise time (jointly with slips). However, here too, rise times per segment are restricted to the values 30–150 s in steps of 30 s; uncertainties about rise times and slips are not computed either. More recently, Melgar & Bock [6] performed a linear deterministic inversion of rupture velocities, as well as an indirect inversion of rise times using multiple time windows of 10 s rise times. Regularization is achieved by spatial and temporal smoothing. Again, uncertainties about slips, rupture velocity and rise times are not computed, but could be large (as we see for some segments in the current paper); this approach cannot account for nonlinear effects and does not quantify uncertainties in the solution.

Bayesian inversion frameworks for tsunami inversion have been carried out recently [11,12]. These approaches, in principle, thoroughly quantify uncertainties in the inversion. Bletery *et al.* [11] considered a discrete set of nine different rupture velocities for the 2004 S-A in the relatively narrow range 2.4–3.2 km s^−1^ and did not account for rise times; they nevertheless noticed that the uncertainties associated with the rupture propagation are significantly larger than the uncertainties on the measurements. Giraldi *et al.* [12] studied the Chile 2010 event, and inverted the latitude and longitude of the epicentre, the strike angle and the slip along the fault, but neither rupture velocities nor rise times. To accelerate inversion, they employed surrogate modelling using polynomial chaos (see [13–15] for other Gaussian process surrogate models of tsunami models). We demonstrate in this paper that a nonlinear inversion of all the kinematic parameters, along with uncertainty assessments, can be achieved using a stochastic inversion approach.

The multi-objective evolutionary method [16] employed here treats the measurements (Jason-1 and T/P SLA) and FF source model parameters (slips, rupture velocities and rise times) as diffusive stochastic processes (see appendix A). Paucity of data and modelling deficiencies impart to the posterior probability distributions of the parameters a multimodal structure that reflects multiple solutions. The stochastic framework offers a natural and rational means to account for multiple solutions, hence simultaneously quantifying their uncertainties. By imposing minimal *a priori* constraints and thus according primacy to the measured data, our work showcases a hitherto unreported large spread in most of the recovered parameters. This in turn calls for a fresh modelling approach by incorporating additional physics related to the dynamics of material discontinuities and heterogeneities such as large sediment cover [17] that are present notably in the northern part of the rupture.

## 2. The measurement: wave height from satellite altimetry

In this work, data from satellite altimetry are employed as the measurement to invert for the rupture parameters. Satellite altimetry measures the same quantity as the tide gauges, i.e. wave height *η*. Altimeters employ microwave radar sensors to measure the distance between the sensor and the ocean surface. Proper corrections, e.g. as employed in the Radar Altimeter Database System (RADS), provide a way to extract the SLA from the altimeter data. Four altimeter-equipped satellites, *viz*. Jason-1, The Ocean Topography Experiment-TOPEX/Poseidon, Environmental Satellite (ENVISAT) and GEOSAT Follow-On (GFO), mapped the ocean in the temporal window 114–544 min post 2004 S-A event origin of 26 December 2004 00.58.53 UTC [4]. SLA levels of considerable amplitudes (approx. 0.7 m) were recovered from the signals after post-processing. This fact makes the 2004 S-A the first event where satellite altimetry enabled delineation of such strong signals for tsunamis in deep ocean.

SLA data for Jason-1 and T/P have been used in this work because they captured the leading wavefront of the tsunami in the deep Bay of Bengal region. The tsunami was detected in the time intervals [1 h 54 min,2 h 3 min 6 s] and [2 h 1 min,2 h 10 min] by Jason-1 (Phase a | Cycle 109 | Pass 129) and T/P (Phase b | Cycle 452 | Pass 129), respectively. The corresponding satellite track intervals can be approximated by the line segments between (83.11°E,7.36°S) and (92.98°E,19.2°N) for Jason-1 and (82.16°E,6.06°S) and (91.98°E,20.26°N) for T/P (figure 1). The early onset time of anomaly detection (approx. 2 h post event origin) is crucial for detecting the primary wavefront of the tsunami. The location of the satellite tracks in deep Bay of Bengal is the key to excluding coastal reflections and dispersive effects. This precludes the need for better resolved shallow water/coastal bathymetry data and dispersive wave propagation models. Moreover, Jason-1 and T/P are tandem satellites. This brings in a form of redundancy in the SLA datasets from the two satellites as evident in the satellite track of Jason-1 being parallel to that of T/P. Also, the orientation of both satellite tracks is near-parallel to the entire length of the rupture (approx. 1400 km). As in addition the SLA capture duration are commensurable (546 s for Jason-1 and 540 s for T/P) with the entire rupture duration (approx. 600 s), we can afford to resolve the rupture velocity and rise times on the scale of the FF segmentation, instead of assuming them to be uniform, as is usually done. The altimeter accuracy (approx. 4 cm), temporal (approx. 1 s) and spatial (approx. 27 km≈15′) along-track resolution and quality post-processing of altimetry data by RADS are other vital features favouring the use of SLA data for inversion.

The SLA data are obtained from RADS with default post-processing that performs many corrections on the raw SLA data, *viz*. orbital, tropospheric, ionospheric, barometric, tidal, geoidal, backscattering, etc. Consequently, there exist gaps in the downloaded SLA data where the raw data do not meet the strict requirements of RADS error budgets. We denote by *t*_*J*_ and *t*_*T*_, the discrete set of time instants and by ***x***_*J*_ (and ***x***_*T*_), the corresponding points on the satellites Jason-1 and T/P (*J* and *T*) tracks where SLA data are available and valid. The number of valid SLA measurements are *n*_*J*_=456 and *n*_*T*_=332 for Jason-1 and T/P, respectively. The SLA data contain spatially and temporally localized oceanographic and meteorological effects that are not removed by RADS post-processing. The multi-satellite time-spatial interpolation (MSTSI) method [18] is used for additional post-processing to extract the tsunami signal (figure 1). These corrections remove some of the uncertainties associated with the SLA data and render it more trustworthy for the inversion. We denote the discrete set of SLA values post-MSTSI correction by *η*_*J*_ and *η*_*T*_, respectively. Neither are additional points added to the SLA data via interpolation (in order to give due primacy to measured data) nor is any relative weighing factor used between the two satellite data. Quite a few discrepancies were found between the SLA data and computed waveforms for the 2004 S-A event even with model enhancements [4]. This study also pointed to the need for a more complex source model as a probable solution. We describe our enhanced source model in the next section.

## 3. The forward models: seabed displacement to the tsunami

### (a) Seabed displacement: kinematic description

Information on the spatial distribution of coseismic displacement along the earthquake rupture is essential for the simulation of the consequent tsunami. The prescription of the coseismic displacement (especially its vertical component), which acts as the initial condition for the tsunami propagation model, significantly impacts the numerical computation of the tsunami wave. As the time scale of the tsunami life cycle is much larger than the rupture process, the dynamics of the rupture are usually assumed to have minimal effect on the tsunami wave. Consequently, the final vertical seabed displacement is equated with the initial sea surface change (or initial conditions) for the tsunami propagation model. For realistic scenarios (e.g. for slow ruptures as reported for the Andaman segment [1,9]), it is more appropriate to endow the source with a kinematic description. Crucially, the incorporation of dynamic parameters for inversion of the tsunami source helps us understand the physical processes underpinning the rupture. As we will see later, even though the inclusion of the dynamic parameters in the rupture may only have a small effect on the far-field tsunami wave, they do have appreciable implications on the near-field (far- and near-field imply remoteness from or proximity to the rupture, respectively). Since mathematical models for dynamic rupture are difficult to develop and generally not available, we make some simple assumptions on the static displacement field to arrive at its dynamic counterpart.

The rupture is modelled as a dislocation in the material along which it is cut and displaced. Building on Volterra's theory of dislocations, Steketee [19] modelled the coseismic displacement as a solution to the seismic dislocation (i.e. rupture) in an elastic medium. Okada [20] presented explicit closed-form analytical formulae for calculating the surface displacement due to a constant dislocation (or slip) across a rectangular fault patch in a semi-infinite isotropic homogeneous medium. We employ this Okada solution to construct the static displacement field denoted by ***u***(***x***), where ***x*** is a point in the computational domain *Ω*. Towards this, a FF segmentation is constructed by discretizing the continuous rupture into a finite number (*n*_F_) of rectangular segments (or sub-faults). A segment (say *i*, where *i* varies from 1 to *n*_F_) is characterized by 10 parameters, seven of which define its geometry *viz*. its length *l*^*i*^, width *w*^*i*^, depth *d*^*i*^, strike *θ*^*i*^_s_, dip angle $\theta_{\delta }^{i}$ and geodetic coordinates $\left(x_{o}^{i},y_{o}^{i}\right)$ of its origin. Another two parameters, the rake angle *θ*^*i*^_*r*_ and slip *S*^*i*^ describe the direction and magnitude of the dislocation on the segment. Mechanical characterization of the segment's medium is carried out by the last parameter *ν*, its Poisson's ratio. All the parameters are assumed to be constant within the segment. The displacement for a given slip is linearly related to the Green's function, which hence only needs to be evaluated once for each segment with a unit slip input. For simplicity, we denote the expressions for the Green's function operator in [20] as O and express the static displacement field resulting from segment *i* as ***u***^*i*^(***x***):

3.1 $$u^{i}(x)=S^{i}O(x,\nu ,l^{i},w^{i},d^{i},\theta_{s}^{i},\theta_{\delta }^{i},\theta_{r}^{i},x_{o}^{i},y_{o}^{i}).$$

Let (⋅)_*z*_ denote the vertical or *z*-component. The total vertical displacement field *u*_*z*_(***x***), which is the primary cause for tsunami genesis, is expressed as the linear superposition of vertical displacements resulting from all the *n*_F_ segments,

3.2 $$u_{z}(x):=\sum_{i=1}^{n_{F}}u_{z}^{i}(x)=\sum_{i=1}^{n_{F}}S^{i}O_{z}^{i}(x,\ldots ,y_{o}^{i}).$$

Now, the static displacement ***u***^*i*^(***x***) is used to construct the dynamic displacement ***u***^*i*^(***x***,*t*) resulting from segment *i* following [7]. To construct this time evolution, two more parameters, the rise time $T_{R}^{i}$ and the rupture velocity $V_{R}^{i}$, are introduced per segment. These parameters are also assumed to be constant within each segment. This key enhancement also renders the source more complex than the simple (and widely used) case where a single rupture velocity and rise time are assigned to the entire rupture. The rupture velocity provides the segments with a temporal ordering corresponding to the propagation of the rupture. The time instance when segment *i* initiates rupturing is denoted by $t_{o}^{i}$. In the present FF model, $t_{o}^{1}=0\;s$ because rupture starts with the first segment. The rupture velocity is related to the rupture initiation instances of adjacent segments (say *i* and *i*+1) as

3.3 $$V_{R}^{i}=\frac{l^{i}}{t_{o}^{i+1}-t_{o}^{i}}.$$

As originally proposed by Hammack [21], the maximum displacement is assumed to be attained in a finite rise time $T_{R}^{i}$. In other words, ***u***^*i*^(***x***,*t*) goes from zero to the maximum coseismic static displacement ***u***^*i*^(***x***) in the time interval $\left[t_{o}^{i},t_{o}^{i}+T_{R}^{i}\right]$. A simple way to carry out this transformation is by multiplying ***u***^*i*^(***x***) with the seabed evolution or rise time function such that the temporal and spatial dependence are mathematically separated. Many rise time functions are available, e.g. the instantaneous (Heaviside), piecewise linear [10], exponential and trigonometric functions [7], or more complex ones [22]. We select the trigonometric rise time function R(⋅,⋅) defined by

3.4 $$R(t,\tau ):=H(t-\tau )+\frac{1}{2}H(t)H(\tau -t)\left(1-\cos\left(\pi \frac{t}{\tau }\right)\right),$$

with H(⋅) denoting the Heaviside step function. This selection is *ad hoc* but, in our problem, the satellite track SLA is relatively insensitive to the particular shape of the rise time function, compared to the rise time itself. The dynamic displacement ***u***^*i*^(***x***,*t*) due to the dynamic behaviour of the rupture in segment *i* is hence defined as follows:

3.5 $$u^{i}(x,t):=u^{i}(x)R(t-t_{o}^{i},T_{R}^{i}).$$

Similar to (3.2), the vertical seabed displacement history *u*_*z*_(***x***,*t*) due to the entire rupture is the linear superposition of individual dynamic displacements resulting from all the *n*_F_ segments,

3.6 $$u_{z}(x,t):=\sum_{i=1}^{n_{F}}u_{z}^{i}(x,t)=\sum_{i=1}^{n_{F}}S^{i}O_{z}^{i}(x,\ldots )R(t-t_{o}^{i},T_{R}^{i}).$$

Thus, the vertical seabed displacement evolves from 0 at rupture initiation time $t_{o}^{1}$ to its final value at the rupture termination time. The rupture termination time or the total time taken by the rupture may vary depending on the relative values of segment initiation and rise times. Normally, it is given by $t_{o}^{n_{F}}+\max(T_{R}^{n_{F}},l^{n_{F}}/V_{R}^{n_{F}})$, the time taken by either the rupture to reach the end of the last segment or the dynamic displacement of the last segment to fully evolve, whichever is greater. In cases where other segments may have relatively larger rise times, the rupture termination time is defined as the time instant when the dynamic displacements due to all the segments have reached their final values.

Various FF segmentations have been employed in the research community for the 2004 S-A event. These have been discussed and compared [23,24]. Here, we use the 14-segment FF model along the Sunda trench from Hirata *et al.* [9] to represent the rupture due to its quality and parsimony. The epicentre is located in (3.3°N,95.68°E), the first segment at a depth of 30 km. This FF model is used in [9] and in a slightly modified form in [25] to invert for the tsunami source from satellite altimetry. It also appears in another form in [26] to jointly invert for the tsunami source from tide gauge and satellite altimetry. The FF parameters except the rupture parameters are fixed with values taken from table 1 of [9]. Notably, *l*=100 km, *w*=150 km, *d*=10 km and *θ*_*δ*_=10° for all the segments. The uniform dip angle of 10° lies between the Harvard centroid-moment-tensor solution of 8° [1] and 10°–12° observed for the aftershocks by the ocean-bottom seismographic network [27,26]. This along with the up-dip depth of 10 km gives a down-dip depth of 36 km. Principal stress axes of the aftershocks from thrust events are used to arrive at the rake angles [9,26]. The extent of the locked fault zone in the Sumatra subduction zone identified by coral growth and GPS assessments are used to constrain the down-dip width to 150 km [9,28].

To complete the description of the rupture, we define the seismic moment *M*_0_ and moment magnitude *M*_*W*_ [29,30] as

3.7 $$M_{0}:=\sum_{i=1}^{n_{F}}\mu l^{i}w^{i}S^{i}$$

and

3.8 $$M_{W}:=\frac{2}{3}(\log_{10}M_{0}-9.1),$$

with *μ*=3.5×10^10^ N m^−2^ [29] being its modulus of rigidity.

### (b) Tsunami: generation and propagation

The seabed displacement triggers a possible tsunami: the released energy propagates as small waves in deep ocean but may attain catastrophic amplitudes in shallow water due to amplification and eventually produces potentially large coastal inundations. We employ VOLNA [31] to model the generation and propagation of the tsunami wave. VOLNA is a well-validated finite volume solver of the non-dispersive nonlinear shallow water equations (NSWEs) on Cartesian coordinates:

3.9 $$H_{t}+\nabla \cdot (Hv)=0$$

and

3.10 $${\left(Hv\right)}_{t}+\nabla \cdot \left(Hv⊗v+\frac{g}{2}H^{2}I_{2}\right)=-gH\nabla h,$$

where *h*(***x***,*t*), ***v***(***x***,*t*), *g* and *H*(***x***,*t*) are the dynamic bathymetry, depth-averaged horizontal velocity, acceleration due to gravity and total water depth, respectively. ***I***_2_ denotes the 2×2 identity matrix. The free surface elevation (or SLA), *η*(***x***,*t*) is given by

3.11 η(x,t)=H(x,t)−h(x,t),

where the dynamic bathymetry is the sum of static bathymetry *h*_*s*_(***x***) and the dynamic seabed deformation *u*_*z*_(***x***,*t*):

3.12 $$h(x,t)=h_{s}(x)+u_{z}(x,t).$$

Thus, (3.12) employs the active mode of tsunami generation distinct from the passive mode constituted by instantaneous uplift [32]. Here, *h*_s_ is sourced from ETOPO1 bathymetry (1′-resolution) [33] and *u*_*z*_(***x***,*t*) is the vertical component of the dynamic seabed displacement from (3.6).

The computational domain *Ω* is [82°E,97°E]×[8°S,21°N], which contains the track portions of Jason-1 and T/P satellites where the tsunami was detected (figure 1). A uniform triangular mesh (element size approx. 1.8 km) for the simulations is built upon the ETOPO1 grid using Gmsh [34]. The time-stepping is done approximately every 1.28 s for a total simulation time *t*_s_ of 2 h 12 min, the time by which the satellites have finished traversing *Ω*. The seabed displacement (3.6) is calculated every 15 s from *t*=0 s till the end of the seabed deformation to update the bathymetry (3.12). While reflections from the islands (northeast of the trench) could in principle contribute to the tsunami, the approximately 10 min temporal window of SLA capture by the satellites is free of such subsequent reflections. Similar remarks hold for reflections from the regions adjacent to the Myanmar coast.

## 4. The inverse problem: reconstructing the source parameters

Using a LSWE, the problem of inverting the slip alone from SLA (or tsunami wave height) is widely posed as linear [8,9,26]. In the LSWEs, the SLA and seabed uplift are linearly related, the latter in turn being linearly related to the slip via the Okada solution (3.2). This enables employing schemes like the non-negative least-squares minimization [35]. Unfortunately, inversions for either rupture velocities or rise times are highly nonlinear (4.1). In a linear setting, this amounts to fixing, perhaps arbitrarily, parameters related to the FF array in (3.1), rupture velocities (3.3), rise time function (3.4), rise times (3.5) and crustal rigidity (*μ*) (3.7). Alternatively, these are deduced from *a priori* finite discrete sets. Complex interactions between the static (slip) and dynamic (rupture velocity and rise time) parameters cannot be reflected in such exercises. As a result, employing some form of variance reduction [9] or jackknifing [8] cannot faithfully reproduce the statistical information and hence the uncertainties in the inverted parameters (see also [36] which discusses artefacts in the inverted solution in the context of seismic inversions). In fact, in a problem as complex as this, a significant part of the non-uniqueness could perhaps arise owing to the limitations and inaccuracies in the forward problem, e.g. the deformation model that should ideally account for thermo-visco-plasticity and damage (including fracture) within a highly inhomogeneous and discontinuous medium like sediment deposits. All the above factors highlight the importance of a nonlinear, joint inversion of the source parameters attempted here.

Among the possible options for joint inversion of slips, rupture velocities and rise times, stochastic methods perhaps offer the most rational framework for tackling the ubiquitous noisy character of real-world problems while naturally dealing with non-uniqueness in the inverted parameters. Surveys of inversion for the 2004 S-A event may be found in [23] and examination of various FF models in [24]. Deterministic schemes face possible ill-posedness and lack of physicality or/and extreme sensitivity to regularization. Such regularizations may include imposition of constraints to limit the search space, slip-velocity positivity [37], weak equalization of adjacent segments’ slip [38], uniform slips, average slip via seismic moment/magnitude and others as discussed in [36]. Imposing different constraints may result in multiple solutions fitting a given dataset. A significant step in the nonlinear inverse problem approach was by [5], wherein the heat bath algorithm [39]—a stochastic simulated annealing method—was used to invert for multiple rupture velocities, rake angles and rigidities. However, it employed a brute-force logic within a bounded set wherein a trial solution at any given iteration differed from the last guess by a fixed value. Collapsing a continuum search to one on a discrete set as above may conceal important sources of non-uniqueness in the reconstructions associated with such nonlinear inverse problems. Consequently, this discretization does not allow for proper quantification of uncertainties in the solution.

We employ an inversion approach founded on a stochastic filtering or projection scheme, namely the Change of Measure Based Evolutionary Optimization (COMBEO) [40,16] (see appendix A for a description and notations). COMBEO has been rigorously vetted against around 40 standard benchmark optimization problems while performing better than many existing heuristic methods [16]. In addition, it is backed firmly by the mathematics of the martingale problem [16,41]. It has, for instance, been employed with great success in optical tomography problems where noisy measurements abound and parameter field reconstructions are non-trivial. Cases with experimental data include diffraction tomography [42] and quantitative photoacoustic tomography [43].

### (a) Formulating the nonlinear inverse problem

In this section, we formulate the inverse problem. We designate the FF slips $\left(S={\left\{S^{i}\right\}}_{i=1}^{n_{F}}\right)$, rupture velocities $\left(V_{R}={\left\{V_{R}^{i}\right\}}_{i=1}^{n_{F}}\right)$ and rise times $\left(T_{R}={\left\{T_{R}^{i}\right\}}_{i=1}^{n_{F}}\right)$ as the rupture variables (or rupture parameters). All the other FF parameters are treated as constants. We simplify the notations in (3.6) to clearly delineate the general functional dependence of the final seabed displacement on the rupture variables, as follows:

4.1 $$u_{z}(x,t)=D(x,t,S,V_{R},T_{R}).$$

The deformation operator D combines the effects of O, the generation of the static seabed displacement (3.1) and R, its transmutation into the dynamic counterpart (3.5). While D is linear in *S*^*i*^ owing to the linearity in the Okada expression (3.1), it is highly nonlinear in $V_{R}^{i}$ and $T_{R}^{i}$ as manifest in (3.5), (3.4) and (3.3). Turning to the tsunami propagation, we consolidate (3.9)–(3.12) into the tsunami operator T to get

4.2 $$\eta (x,t)=T(x,t,u_{z}(x,t)).$$

As is evident from (3.10), T is nonlinear in *u*_*z*_. We substitute (4.1) in (4.2) to portray the dependency of *η*(***x***,*t*) on the rupture variables that are hidden in *u*_*z*_(***x***,*t*),

4.3 $$\eta (x,t)=T(x,t,D(x,t,S,V_{R},T_{R})).$$

From (4.3), it is clear that T is also nonlinear in *S*^*i*^, $V_{R}^{i}$ and $T_{R}^{i}$.

Coming to the measurements, we concatenate the individual satellite SLAs, $\eta_{J}\in R^{n_{J}}$ and $\eta_{T}\in R^{n_{T}}$ to constitute the SLA measurement vector $\eta_{M}\in R^{n_{M}}$ (with *n*_M_=*n*_J_+*n*_T_=788),

4.4 $$\eta_{M}=[\eta_{J}\;\eta_{T}].$$

The corresponding combined satellite track locations and tsunami detection time instances are similarly constituted by ***x***_M_=[***x***_*J*_ ***x***_T_] and ***t***_M_=[***t***_*J*_ ***t***_T_], respectively. The computed counterpart $\eta_{C}$ of the measurement vector *η*_M_ (4.4) is formed as a restriction of the free surface elevation (4.3) over the satellite tracks. For clarity, we suppress ***x*** and *t* and render (4.3) into a composition of the two forward models (D, T) characterized by the measurement operator M that maps the rupture variables to the computed measurement (A 2),

4.5 $$\eta_{C}=T\circ D(S,V_{R},T_{R}){|}_{\left(x,t)=(x_{M},t_{M}\right)}=M([S\;V_{R}\;T_{R}]).$$

The argument of M is the vector of parameters ***p*** that are to be inverted (A 1). In (4.5), the most general case of inversion is when all the rupture variables are inverted for. In line with the discussions above, although M may be considered weakly nonlinear in slips, it is strongly nonlinear in rupture velocities and rise times. The parameters ***p*** and measurements *η*_M_ are treated as stochastic processes in COMBEO which evolve along a time-like axis (hence, the subscript *τ* in (A 1)–(A 2)). The measurement-prediction misfit (or the innovation in the filtering parlance) *e* is defined as the signed error between the actual observation *η*_M_ and its computed counterpart $\eta_{C}$,

4.6 $$e(p)=\eta_{M}-\eta_{C}.$$

A deterministic route to solve an inverse problem seeks to find $p_{\min}$ that minimizes a suitable norm of the vector-valued *e*(***p***) (say, ∥*e*(***p***)∥_2_). On the other hand, COMBEO tries to find a joint distribution of the rupture variables that drives each component of *e*(***p***) to a **0** mean Brownian motion, the stochastic equivalent of the deterministic 0. Thus, COMBEO can handle a multivariate objective function as it works on a vector of signed errors instead of a positive scalar (i.e. a norm). Indeed, the signed version of the misfit is also better than its unsigned version (absolute value), because the former contains more information about the error. The vector-valued misfit enables the maximum possible assimilation of the available information, compared to the common inversion schemes where the misfit is flattened into a scalar error. As a result, COMBEO typically achieves a better fit for the whole duration of the observed SLA than other inversion methods. Finally, we note that the inversion of the parameters from 14 segments is aided to a great extent by the availability of 788 high-quality SLA measurements on satellite tracks that are near-parallel to the entire rupture.

### (b) Choice of parameter space for inversion: Cases A, B and C

We first perform a sensitivity study of the kinetic parameters. The aim is to explore the sensitivity of the satellite track SLA to rupture velocity and rise time.

**Figure 2.**
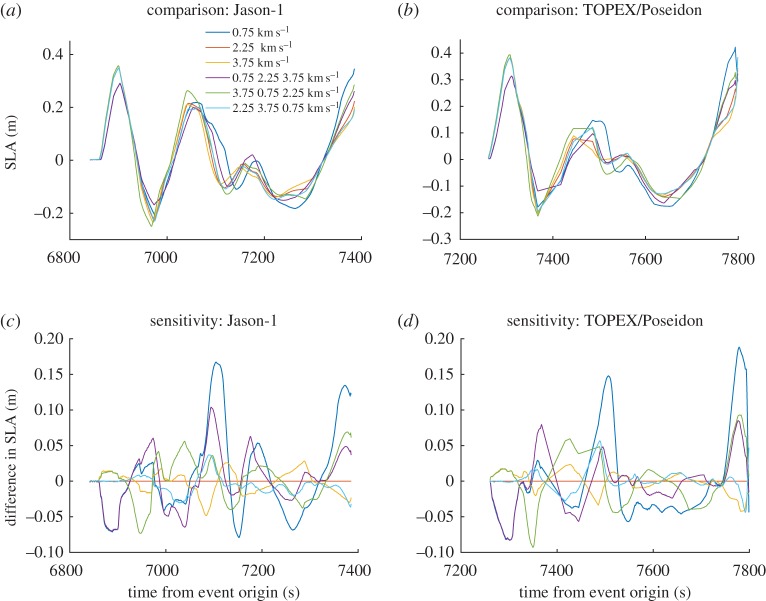
Satellite track SLA (*a*) Jason-1 and (*b*) T/P for different rupture velocities assigned to different segments. Difference between these cases and the waveform computed with 2.25 km s^−1^ as the baseline, (*c*) Jason-1 and (*d*) T/P.

The sensitivity of satellite track SLA to the rupture velocity is displayed in figure 2. The slips and rise times are fixed at 15 m and 100 s for all the segments. The rupture velocities are chosen as 0.75, 2.25 and 3.75 km s^−1^, uniformly throughout the 14 segments of the fault. Then, the segments 1–5, 6–10 and 11–14 are assigned different rupture velocities in a cyclic manner (see legend in figure 2). The first and second columns show the results on tracks of satellites Jason-1 and T/P, respectively. The first row shows the tsunami wave height (*η*). The second row shows the difference in tsunami wave heights with the one corresponding to 2.25 km s^−1^ as the baseline.

**Figure 3.**
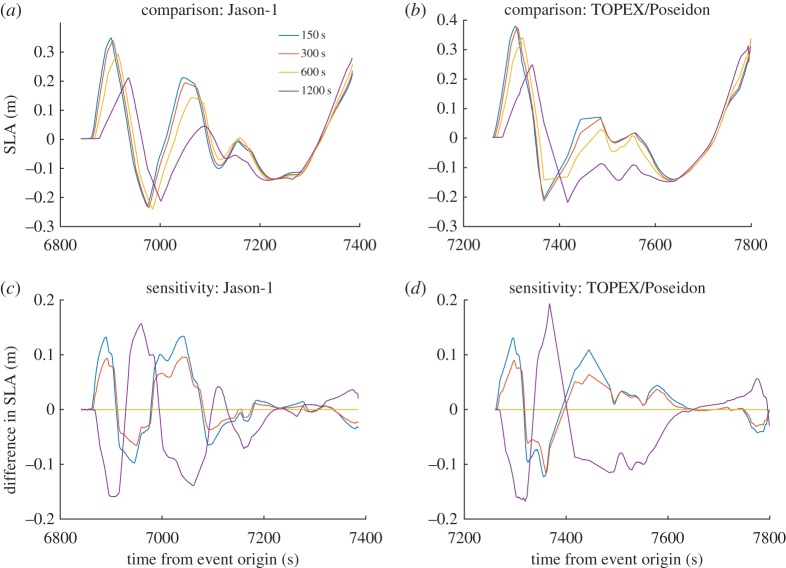
Satellite track SLA (*a*) Jason-1 and (*b*) T/P for different rise times assigned to different segments. Difference between these cases and the waveform computed with 600 s as the baseline, (*c*) Jason-1 and (*d*) T/P.

The sensitivity of satellite track SLA to the rise time is displayed in figure 3. The slips and rupture velocities are fixed at 15 m and 2.25 km s^−1^ for all the segments. The segments are allocated rise times of 150, 300, 600 and 1200 s uniformly. Here, the second row shows the difference in tsunami wave heights with the one corresponding to 600 s as the baseline. The differences are of similar magnitude when compared with those for rupture velocities.

Now, with the purpose of bringing out the complex interplay between the rupture variables, we consider three cases of inversion—Case A (inversion of slips alone), Case B (slips and rupture velocities) and Case C (slips, rupture velocities and rise times).

*Case A*: This simplest case involves inversion of the slips alone while fixing the values of the rupture velocities and rise times. For this case, the right-hand side of (4.5) reduces to M([S]) (with ***p***=***S***). This is the case usually encountered in the literature [9,26]. The inversion may be construed as linear in the slips, provided one neglects the effect of the nonlinear terms in the NSWE (3.10) for deep ocean. Hence, although the Green's function approach (or a variant with linearization about the mean slip for the NSWE [5,44]), in practice, could be employed here, we do not pursue that route to maintain uniformity in simulations for the subsequent nonlinear scenarios, *viz.* Cases B and C. The number of parameters to be inverted (*n*_*P*_) is equal to the number of slips or the number of segments in the FF model (*n*_F_=14). Reasonable values of the rupture velocities and rise times, consistent with other findings in the literature, are fixed at 2.5 km s^−1^ and 60 s, respectively, in all the segments.

*Case B*: Here, the rupture velocities are posed as additional unknowns, which increases the complexity of the inversion (i.e. now, *n*_*P*_=2×*n*_F_=28). This implies that the inversion algorithm independently searches for the 14 slips and 14 rupture velocities, while all the 14 rise times remain fixed at 60 s. Lorito *et al.* [5] employed with success the Green's functions for such a scenario using the NSWE and inverted for the 18 slips and four rupture velocities for a 9×2 segment FF model. But unlike our case where each of the 14 segments is allowed an individual rupture velocity, Lorito *et al.* [5] partitioned the FF model into four subfault groups, each rupturing together as a unit with the same rupture velocity. More importantly, in this work the active mode of tsunami generation is employed [32]. This allows dynamic rupture to be more consistently manifested in both the dynamic seabed uplift (3.12) and consequently in the SLA instead of shifting/delaying the Green's functions [5]. The right-hand side of (4.5) reduces to $M([S\;V_{R}])$ (with ***p***=[***S*** ***V***_R_]) for this case.

*Case C*: We include the 14 rise times also to be independently searched by the inversion algorithm inflating the parameter space to 42 dimensions (i.e. now, *n*_P_=3×*n*_F_=42). As is evident in (3.5), the rise times bring in further nonlinearity that precludes applying Green's functions or linear inversion schemes. To our knowledge, this is the first time that such an inversion attempt has been made for a megathrust event.

Minimal *a priori* constraints are imposed in the form of bounds within the inversion algorithm to limit the search in the parameter space. The bounds for the slips, rupture velocities and rise times are (0, 100) m, (0.4, 4) km s^−1^ and (30, 300) s, respectively, in line with the current physical understanding. The initial guess for these parameters at the start of the search are, respectively, 10 m, 2.5 km s^−1^ and 60 s. Parameters at any given iteration in COMBEO are perturbed by an a priori noise ***β***, as the parameter evolutions follow stochastic differential equations (SDEs) (A 1). Nevertheless, the exploration rendered by the process noise must also not push the solution too far away from the neighbourhood of its current optimal solution. Therefore, the entries in the parameter noise covariance matrix $\Sigma_{\beta }\Sigma_{\beta }^{T}$ need to be selected in a manner so as to not render the recursive procedure divergent. Preferably, they should be at least an order of magnitude lower than the solution. Here, we fix $\Sigma_{\beta }\Sigma_{\beta }^{T}={\left(0.1\right)}^{2}I_{14}\;m^{2}$ in Case A with ***I***_14_ denoting the 14×14 identity matrix. For Cases B and C where rupture velocities and rise times are inverted, the corresponding diagonal blocks of $\Sigma_{\beta }\Sigma_{\beta }^{T}$ are populated by ${\left(4\right)}^{2}I_{14}\;m^{2}\;s^{-2}$ and ${\left(0.1\right)}^{2}I_{14}\;s^{2}$, respectively.

The uncertainty in the measurement is another important, yet often overlooked factor, which is to an extent accounted for in COMBEO by the measurement noise ***γ*** (A 2). The entries in the measurement noise covariance matrix ***Σ***_***γ***_***Σ***^*T*^_***γ***_ must be relatively small in comparison to $∥\eta_{M}∥$ so that ***m***_*τ*_ stays in the vicinity of *η*_M_. Here, we fix $\Sigma_{\gamma }\Sigma_{\gamma }^{T}={\left(10^{-3}\right)}^{2}I_{788}\;m^{2}$, where ***I***_788_ denotes the 788×788 identity matrix.

**Figure 4.**
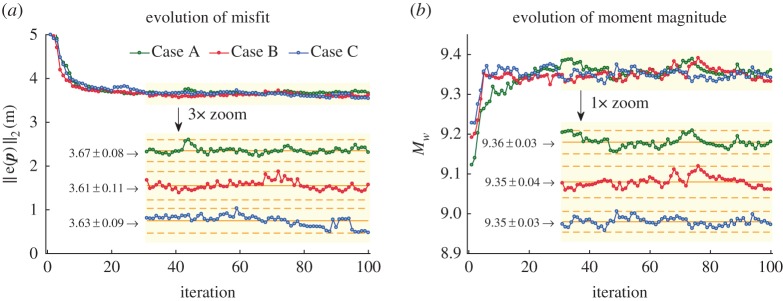
Evolution of (*a*) the *l*_2_-norm of the multi-objective error *e*(***p***) and (*b*) moment magnitude *M*_*W*_. The error norm (and moment magnitude) plateaus around 30 iterations (*shaded*) after which very small fluctuations are noticed. See the corresponding SLA waveform matches in figure 5.

In the presence of multimodality in the posterior distribution of the parameters (i.e. multiple possibilities for the solution), obtaining an acceptable solution is based on a judicious combination of the local and global search features available in COMBEO. However, being a Monte-Carlo scheme, implementing COMBEO is a computationally intensive endeavour requiring repeated numerical evaluations of VOLNA. The search is continued until the misfit attains the statistical properties of the measurement noise (i.e. a zero-mean stochastic process with independent increments), but practically it is halted when the misfit error plateaus or becomes less than a tolerance value (figure 4). The stopping criterion for the algorithm varies with the problem at hand, but in general, a trial and error procedure is required to determine the total number of iterations (here, we stop at 100 iterations).

**Figure 5.**
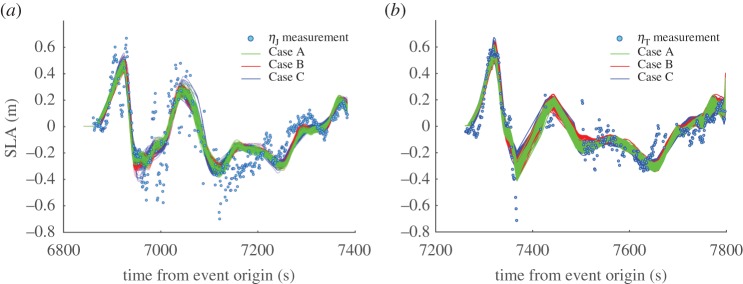
Waveform fits for computed (*a*) Jason-1 and (*b*) T/P SLA after the error norm plateaus (i.e. iterations 31–100; see figure 4).

The search provides a natural means to the empirical quantification of the various uncertainties involved. Indeed, the positions of the particles along the search, after convergence has been obtained, represent the intrinsic uncertainty about the solution of the inversion problem. The number of particles (i.e. ensemble size *n*_*E*_) was limited to five because it provided satisfactory results with no need to increase further.

### (c) Uncertainty propagation: near-field effects

**Figure 6.**
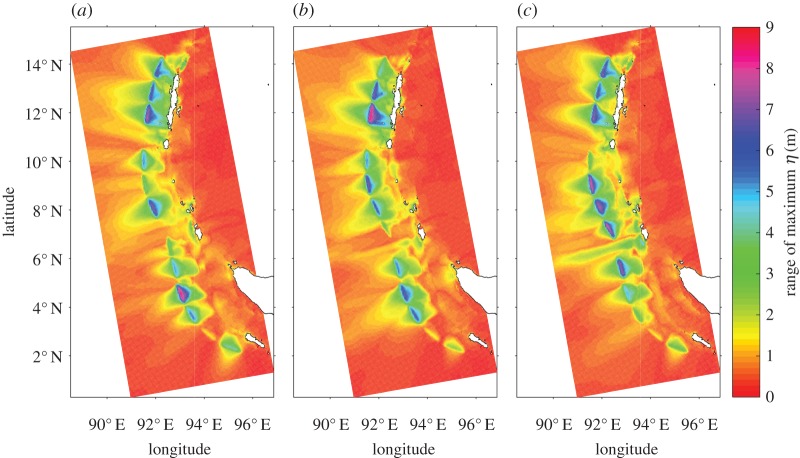
Range of maximum wave height. The value at each point in the plot shows the range of the maximum wave heights at that point resulting from forward propagation of the 70 parameter sets (i.e. from iterations 31–100). The near-field effects of the different parameter distributions: (*a*) Case A, (*b*) Case B and (*c*) Case C. Observe significant differences in the tsunami wave height throughout the rupture length between the three cases.

The effect of inverted parameter distributions on near-field tsunami waves is showcased by propagating these uncertain input parameters through the forward model (figure 6). The near-field basin comprises the area of the graph shown in figure 1, a region encompassing the whole of the rupture. The parameters from iterations where the error reaches steady state (i.e. 31–100) are employed for this exercise. This is primarily done to estimate the hazard implications of the different source parameters, especially the rupture velocity and rise time.

The maximum tsunami height at every point of the near-field basin (figure 1) is computed for each of the 70 parameter values (i.e. corresponding to iterations 31–100). The range of these 70 maximum wave height values (i.e. the difference between the maximum and minimum of the set of 70 maximum wave heights) is plotted in figure 6. We note that differences of several metres in these ranges can be spotted across Cases A, B and C. Different inversions can produce not only vastly different tsunami predictions, but also ranges about these predictions that can be significantly dissimilar. It highlights the need to make an informed choice about the parameter setting employed for the inversion.

## 5. Discussion

In all the three cases, the inverted parameters are reported after the *l*_2_-norm of the multi-objective error attains steady state at around 30 iterations, beyond which only very small fluctuations are noticed (figure 4). Similar observations apply to the moment magnitudes and seismic moments which attain steady state at about 9.35 and 13.5×10^22^ Nm, respectively. The effect of realizing a steady state in the error norm is clearer in the corresponding fits between measured SLA and computed waveforms for both the satellites (figure 5). The waveforms computed after 30 iterations are seen clustered about the measured SLA for all the three cases. Careful observation reveals that the computed waveforms tend to match better the measured SLA waveform over certain intervals on inclusion of more parameters for inversion. Thus relatively speaking, the envelope of computed waveforms for C (joint inversion of slips, rupture velocities and rise times) enables a better reconstruction than B (joint inversion of slips and rupture velocities), which in turn provides a better fit than A (inversion of slips alone). Fig. 6 in [11] displays similar variations in the inverted SLA waveforms about the measured SLA, although without the use of rupture velocities and rise times. Their inverted slips along the segmentation are overall consistent with ours, but also show some dissimilarities, again possibly due to a different parameter setting. It is reassuring that using a different route, our results agree with the other approaches in more restricted settings.

**Figure 7.**
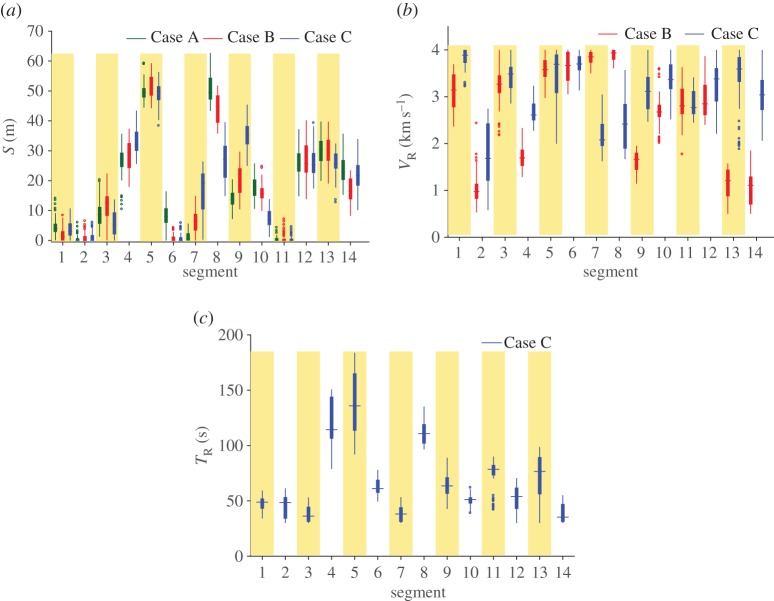
Empirical distributions of inverted (*a*) slips, (*b*) rupture velocities and (*c*) rise times. Case A, inversion of slips only; Case B, joint inversion of slips and rupture velocities; Case C, joint inversion of slips, rupture velocities and rise times.

In the absence of previously reported exercises on similar lines, a comparative assessment of the iterative evolution of parameters, errors and other derived quantities (like *M*_0_ and *M*_*W*_) reported here is not possible. The fast and near-monotonic decay of the error norm results from the superior form of the exploration–exploitation trade-off embedded in COMBEO and owing to a multi-objective search. Variances in the parameters captured by the method in all the three cases are quite significant, even though the respective errors have attained a steady state (figure 7). The variances are also different for each case.

For none of the cases, the slips reach the prescribed upper bound of 100 m. High mean slips of 30–60 m are consistently observed in segments 4 and 5. The variances are visibly quite different between the three cases, especially in segments 7–9. Mean slips of 20–30 m are also consistently seen in the northernmost segments 12–14 where the rupture terminates. Although unusually high, they are supported by a good fit in the northern end of the waveforms. Interestingly, coseismic land level changes observed both from geodetic and field evidence also suggest variations along the rupture zone with its northern terminus (segment 14) showing a relatively larger vertical offset [45]. Overall, the slips here are relatively higher than previously reported, e.g. table 2 in [23] and in [24]. This also results in the marginally higher values of *M*_0_ than that reported in the former (5.7–11×10^22^ Nm). The inflated slips (and *M*_*W*_) are compensated values (compensated by the inversion algorithm). At least one previous work [25] explains, in similar terms, a higher slip reported therein. We identify two reasons reported in the literature that shed light on the slip inflation. First, based on the study in [17], we estimate a 60% increase locally in the deformation uplift due to the presence of sediments. This could reduce the compensation by the algorithm and result in proportionately lower slips. Second, Shearer & Bürgmann, in their comments on table 2 of [23] in the context of seismic and geodetic inversions for the 2004 S-A source, explain a halving of the moment magnitude (or a 0.2 decrease in *M*_*W*_) due to an increase in the dip angle assumed. We do not change our assumptions to conform to an *M*_*W*_ in the range of 9.1–9.3, because one of the main aspects of the work is to highlight the inadequacies of the physical models. Thus a closer match could have been enforced, e.g. by assuming a steeper dip angle in the FF geometry [23], a strategy avoided here. Similarly, the terminal high slips could also have been obviated by imposing the *a priori* constraint of zero slip in the rupture termination zone. In other words, we point to an important underlying source of uncertainty which is liable to be glazed over in the event of matching the *M*_*W*_ or imposing further constraints. A closer examination of the SLA reveals unmatched fluctuations that could be ascribed either to coarse segmentation or deficiencies in the model.

Large uncertainties are also seen in the rupture velocities although they are curtailed by the prescribed upper bound of 4 km s^−1^. Significant is the discrepancy when the rise times are included in the inversion, particularly for segments 7–9 and the last two terminal segments (13 and 14). The variances nearly encompass the range of values given in tables 2 and 3 of [23], i.e. 1.5–4.1 km s^−1^. To the best of our knowledge, there are no existing inversions for rise times using SLA data let alone joint inversions, so comparisons are difficult. The uncertainties in rise times seem relatively larger for segments 4, 5, 8, 9 and 11–13.

Parameter distributions in many segments are clearly skewed, highlighting the non-Gaussianity of the posterior distributions. This is manifested, for instance, in segment seven wherein the nature of skewness changes markedly in the three different inversion cases. Moreover, considerable uncertainties in parameters found here explain the plethora of related values reported previously, clearly mandating a more nuanced interpretation of the results than is typically done.

It is not surprising that the fit (figure 5) looks best in the initial few peaks and troughs for Case C because it uses the maximum number of parameters (with *n*_P_=42) among the three cases. Case B (with *n*_P_=28) is next in line followed by Case A (with *n*_P_=14). From a statistical viewpoint, the errors in the three cases are indistinguishable (figure 4*a*). The Akaike information criterion (AIC) [46,47] may be employed as a statistical information-theoretic measure for the relative quality of the three models (or cases). It should be noted that the AIC cannot be readily calculated here because we do not perform a likelihood-based statistical inference. However, inspired by the match between the maximum-likelihood and least-squares estimates in the case of least-squares inversion, we compute a pseudo information criterion (with an abuse of notation) using the residual sum of squares (RSS). The number of SLA measurements (*n*_M_=788) is taken as the sample size for AIC calculation. The expression for AIC in [47] is then recast as

5.1 $$\mathrm{AIC}=n_{M}\log(e_{\mathrm{agg}})+2n_{P},$$

where *e*_agg_ is an aggregate RSS defined as the sum of squares of the errors for iterations 31–100 (i.e. the stabilized region in figure 5). Ideally, the AIC needs to be calculated for all the instances of the three models under consideration (i.e. 70×3=210 instances in total), but the use of *e*_agg_ enables us to aggregate each case (or model). Owing to the finite sample size of the measurement (i.e. 788), we use the corrected AIC or AIC_*c*_ [47] given by

5.2 $${\mathrm{AIC}}_{c}=\mathrm{AIC}+2n_{M}(n_{M}+1){\left(n_{M}-n_{P}-1\right)}^{-1}.$$

This correction exerts considerable influence when the number of measurements available is small. Here, it does not influence the AIC_*c*_ calculation much (see the column for correction AIC_*c*_-AIC in table 1). The relative quality of the three models is revealed in the Kullback–Leibler (K-L) information ranking (Δ^KL^), calculated as

5.3 $$\Delta_{i}^{\mathrm{KL}}={\mathrm{AIC}}_{i}-{\mathrm{AIC}}_{\min},\;i=A,B,C,$$

where AIC${\;}_{\min}$ denotes the minimum of AIC values across the three cases. Similarly, Δ^KL^_c_, the ranking using AIC_c_, may be defined on similar lines. The value of Δ^KL^ for a model is proportional to the K-L information loss in it relative to the other models. The calculations are listed in table 1. Case A (or Model A) has the minimum AIC_c_ (and AIC) and Case B is close to Case A in the sense of the K-L distance. Case C is relatively far from Cases A and B. At first glance, it seemingly implies that Case C (i.e. inclusion of rise times in the inversion) has no empirical support. But AIC is an information-theoretic measure and may not be able to capture the nuances in the physical modelling of the problem. Rather than just eliminate the rise times from the parameter space, the way forward is to model better the dynamic parameters in the rupture process while incorporating more of the physical phenomena involved.

**Table 1.** Ranking of the three cases using the AIC.

case	*n*_P_	AIC	AIC_c_	AIC_c_-AIC	Δ^KL^	Δ^KL^_c_
fig A	14	5426	5427	1	0	0
B	28	5429	5431	2	3	5
C	42	5465	5470	5	39	44

Finally, we illustrate the impact of uncertainties in rupture velocities and rise times on wave heights. Melgar [6] showed that the impact manifests itself close to the source. We display in figure 6 the range of maximum wave heights that result from the uncertainties in the inversion. We find that, indeed, these ranges are similar in the far field but are appreciably different near the source. We also observe that there are localized patches with larger spreads when dynamic parameters are included: this underscores the necessity to include these dynamic parameters when investigating local hazard footprints. A network of buoys near the source would have provided better constraints for the estimation of the dynamic parameters.

## 6. Conclusion

The rational approach employed in COMBEO, the evolutionary stochastic optimization scheme adopted here, *en route* to a joint nonlinear inversion while also addressing the problems inherent in common deterministic schemes, is perhaps a significant first step forward for earthquake source recovery problems. The rational yet flexible machinery of advanced nonlinear inversion techniques allows for an attempt not only at recovering the dynamic source model of the 2004 S-A event but also at accounting for complex interactions between the static (slip) and dynamic (rupture velocity and rise time) parameters. Instead of the usual variance reduction by incorporating arbitrary *a priori* constraints, the possibility of multiple solutions is retained by imposing a minimal set of constraints. The resulting uncertainties could originate from the limitations (such as uncertainties of epistemic origin) in the physical models and paucity of measurement data. In view of the large variances observed, this study emphasizes the need for better physical models.

A key source of epistemic uncertainty enters the joint inversion due to the paucity of dynamic source (i.e. slip history) models. Usual inversions, where slips alone are inverted, fix the slip initiation and rise time of the segment, thus making the segment slip the only unknown in its slip history. Our amelioration allows a joint evolution of the segment slip, its initiation time (deduced from its rupture velocity) and rise time. A parametrization of rupture dynamics using a rupture velocity and rise time per segment invariably makes them sensitive to the segment geometry, besides the coarse resolution resulting from segment-wise rise times given the infinite frequency spectrum of the actual slip history. Accordingly, our parametrization is a significant development, though not nearly the best possible. As with slips, this uncertainty is stochastically captured and quantified by COMBEO. Consequently, it is pertinent not to lose sight of inadequacies in the existing physical models and lack of suitable source models while interpreting any inversion result, including the findings here. Finally, it is remarkable that although uncertainties in measurement (SLA data) also contribute to the variances, the data suffice to constrain the parameters within the bands reported. This brings into focus the practice adopted here of according due primacy to measurement data over *a priori* constraints. As additional data are incorporated within the scheme, a reduction in parameter variances ought to be indicative of the quantum of additional information contained therein.

An important component in modelling uncertainties emerges from the simplifying assumptions in the Okada model, e.g. isotropy, homogeneity and elastic half-space. The lateral variations in the material property and in the bathymetry are also unaccounted for. Similarly important is the choice of parametrization in the practical utilization of the Okada model which includes the geometry of the FF array defined by its orientation, spatial spread, number of segments and grid spacing [24]. The 2004 rupture lies in the Bay of Bengal, where the ocean floor is overlain by kilometres-thick sediment cover [48] especially near its northern termination where it is up to approximately 5–10 km thick. The nonlinear and non-classical behaviour of this compacted granular media is not reflected in the Okada model. In the absence of such physical models, incorporating spatial distribution of material properties in the finite-element method (FEM) [49,17] only addresses the problem in a limited manner. COMBEO's stochastic compensation for this unaccounted material behaviour may be a reason for (i) the relatively high mean slips throughout the rupture, (ii) the apparently high slips (40–60 m) in segment five and (iii) the high mean slips (20–30 m) in the rupture termination region.

Indeed, although previous works point towards high slips in the northern regions of the rupture, the present study offers a rational confirmation. In all three inversion cases, we also recover high slips in the vicinity of the rupture termination. However, such high slips at the rupture termination are physically unrealistic. We believe this is due to unaccounted contribution from the sediments layer, which is much thicker in the northern regions of the rupture than in the other rupture locations. Precisely, Dutykh & Dias [17] quantify the amplification as a function of the ratio of the sediment thickness to down-dip depth. In the northern regions of the rupture, this calculation results in an amplification of around 60% of the seabed uplift.

Our results are consistent with some of the high slips found 300–500 km away from the initiation of rupture [25]. These may be considered slips that compensate for unaccounted physical factors such as the inelastic crustal deformation. The authors of [1] reported that the northward propagating rupture velocity is about 2 km s^−1^ for the first 745 km, and then slows to 0.75 km s^−1^. We also find a general drop in the rupture velocity at around 600–800 km but not of that magnitude: from close to 4 km s^−1^ to around 2.5 km s^−1^. We also observe an increase in the rupture velocity from 900 km. We also note a complex interplay between the dynamic parameters in the inversion. Additionally, the fact that the sensitivity to dynamic parameters reduces in the far field (where the SLA track is located) may lead to a large range of possible values for these parameters.

The results here may be improved by incorporating various enhancements, e.g. FEM modelling of the source, adopting the spherical coordinate version of the NSWE, high-resolution bathymetry datasets, etc. Likely improvements withal, parameter reconstructions for a problem as complex as this would continue to exhibit significant uncertainty bands. This shows that a stochastic scheme like COMBEO should continue to remain relevant as an effective tool for tsunami source recovery. The characteristics of the uncertainties reported here (e.g. skewness, variance, complex inter-dependencies) strongly suggest the need for developing more sophisticated physical models.

The performance of early warning systems could be, in principle, improved by a rational inclusion of uncertainties in the prediction of hazard footprints. This would require the adoption of stochastic methods as in the present paper. However, deterministic schemes, being much faster than such algorithms, have presently proved more useful in early warning systems; see e.g. a statistical inversion framework for near real-time tsunameter data [50]. We note that recent progress in the field of emulation shows much promise for the acceleration of tsunami models by constructing statistical surrogates of the computer model (see [13] for a computationally efficient method and [15] for the emulation of a landslide-induced tsunami). Accordingly, these methods have the ability to extend the scope and speed of stochastic algorithms for early warnings.

## Figures and Tables

**Figure 8.**
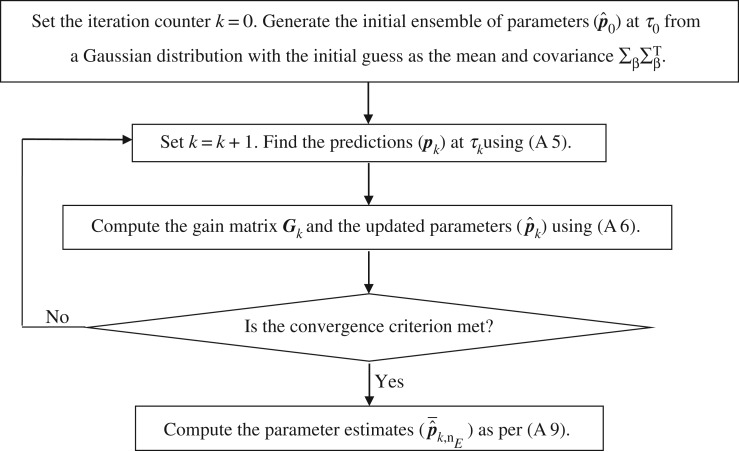
Flowchart for the stochastic evolutionary search algorithm COMBEO.

## Data accessibility

ETOPO1 data are obtained from https://maps.ngdc.noaa.gov/viewers/wcs-client/. SLA data are obtained from http://rads.tudelft.nl/rads/rads.shtml using default post-processing. The codes for COMBEO are available in the companion website of [51].

## Appendix A. Multi-objective stochastic evolutionary optimization

The stochastic optimization employed here is a variant of COMBEO—a change of measure-based evolutionary optimization [16,40,51]. As a necessary first step to solve the problem using COMBEO, the parameters to be inverted, *viz*. the slips, rupture velocities and rise times of the FF segmentation and the measurements, *viz*. the Jason-1 and T/P SLA are treated as stochastic processes (e.g. random walk models in a time-discrete set-up with the optimization iteration counter as the pseudo-time variable) [52]. Such a parametrization of the underlying variables is in contrast with typical Bayesian schemes wherein the unknowns are merely treated as random variables. However, a temporal evolution is demanded by the underlying stochastic calculus [52,41] that leads up to the crucial update step, which forms the bare-bones of COMBEO. Being a Bayesian scheme [53,54], COMBEO provides a natural framework to account for the multiple solutions that may appear in an ill-posed inverse problem [55]. This may be contrasted with the computationally complex regularization procedures employed in usual deterministic schemes to tackle non-uniqueness. The inverted parameters are rendered sensitive not only to such regularization but also to measurement noise, particularly in the presence of sparse measurement data. Thus, where a deterministic scheme is ill-equipped to account for noise, a Bayesian scheme is naturally equipped to properly appropriate the measurement noise into the posterior distributions of the inverted parameters. To bring in more clarity to the steps in the algorithm, we provide a flowchart (figure 8).

A Bayesian scheme, in essence, is characterized by its prediction and update steps which are recursively repeated over iterations [54]. Whereas the prediction step propagates the last updated or initially specified parameters to the current instance, the update step assimilates the currently available measurements so as to render the measurement-prediction misfit into a zero-mean noise process (or, more precisely, a zero-mean martingale such as an Ito integral). Ideally, at any given iteration, the parameter random variable is represented in terms of a finite set of its realizations known as an ensemble. Each realization (or particle) from the prior distribution is weighted according to its corresponding measurement-prediction misfit such that the best fit particle attains the highest weight and so on. Essentially, a typical Bayesian update is given by the product set of the predicted particle and its relative weight. Such an update strategy emphasizes search in locally optimal regions as dictated by a few fit particles and is therefore highly recommended to solve convex optimization problems. Otherwise, a multiplicative update will eventually lead to sample degeneracy as the entire weight gets assigned to a single particle as iterations progress. Such a particle collapse stalls the update and necessitates prohibitively large ensemble sizes to solve high-dimensional inverse problems [56].

In COMBEO, however, such a scenario is averted by converting the multiplicative weights to additive updates which are applied to the predicted particles. This update, in a sense, ‘cures’ particles with low weights by bringing them closer to regions of higher fitness. This additive correction is derived rigorously from the multiplicative update rendered by the underlying Girsanov change of measure [52,41], using the machinery of stochastic calculus (see [40] for a detailed derivation). Thus, the additive updates perform the intended functionality of the multiplicative weights with significantly smaller ensemble sizes as the sample degeneracy issue is no longer a concern [40]. Inherent to the convergence of the iteration procedure in COMBEO is the transformation of the measurement-prediction misfit to a zero-mean martingale. Informally, this would mean that the misfit process becomes mean-invariant to further noisy (i.e. zero-mean) perturbations in the parameter process. This is indeed the stochastic equivalent of the absolute zero (sought in deterministic, quasi-Newton search schemes), around which the first variation of the misfit should be zero. See [41,51,52] for a more formal and detailed exposition of martingales.

The procedure discussed so far may be looked upon as an attempt to solve the filtered martingale problem [57], which at best, is a quasi-local search. The success of an optimization algorithm, on the other hand, depends on striking the right balance between exploiting the optimal information at hand and simultaneously exploring the search space for better solutions. In achieving this, COMBEO uses a filtered martingale approach for local optimization along with a family of perturbation schemes, e.g. coalescence, scrambling and selection, for the global search. Fitted with a stochastic version of directional search that requires no derivative computation, as explained later, COMBEO has been found to be far more successful in solving ill-posed inverse problems as opposed to its counterparts such as particle swarm optimization, differential evolution and covariance matrix adaptation evolution strategies [58,16]. We believe that this is, at least in part, due to the additive nature of the updates and the ability to handle multivariate objective functions without having to reduce them to a scalar functional. In what follows, we give the relevant mathematical equations and formulae that lead up to the prediction–update recursion in COMBEO.

As discussed earlier, a pseudo-dynamical character is imparted to the iterative procedure (even though the parameters and measurements may be inherently static) by introducing a time-like variable *τ* that increases with iterations, i.e. 0=*τ*_0_<*τ*_1_<⋯<*τ*_*k*_<⋯  with the increasing subscript indices denoting the iteration number. Describing the misfits and parameters as stochastic processes in *τ* permits the discrete iterative procedure to be the discretized version of the continuous evolution of a pseudo-dynamical stochastic system. The SDEs that describe the evolution of the parameters and measurements may be written as

A 1 $$dp_{\tau }=d\beta_{\tau }$$

and

A 2 $$m_{\tau }=M(p_{\tau })+\gamma_{\tau },$$

where $p_{\tau }:=p(\tau )\in R^{n_{P}}$ and $m_{\tau }:=m(\tau )\in R^{n_{M}}$ are the stochastic processes corresponding to the parameters (***p***) and measurements (or observations *η*_M_). The number of parameters and measurements are denoted by *n*_P_ and *n*_M_, respectively. We see that $\beta_{\tau }:=\beta (\tau )\in R^{n_{P}}$ and $\gamma_{\tau }:=\gamma (\tau )\in R^{n_{M}}$ are **0** mean Brownian motions with covariances $\Sigma_{\beta }\Sigma_{\beta }^{T}\in R^{n_{P}\times n_{P}}$ and $\Sigma_{\gamma }\Sigma_{\gamma }^{T}\in R^{n_{M}\times n_{M}}$, respectively. In (A 2), $M:R^{n_{P}}\mapsto R^{n_{M}}$ is the measurement operator that maps the unknown parameters through nonlinear forward models to the computed measurements (i.e. $M(p)=\eta_{C}$). Also, ***m***_0_ and ***γ***_0_ comprise the actual measurement (i.e. the SLA data *η*_M_) and the noise content in it, respectively. The entries in the measurement noise covariance matrix ***Σ***_***γ***_***Σ***^*T*^_***γ***_ must be relatively small in comparison to $∥m_{0}∥$ so that ***m***_*τ*_,*τ*>0 stays in the vicinity of ***m***_0_. Discretizations of (A 1) and (A 2) give the following set of equations for the interval (*τ*_*k*_,*τ*_*k*+1_]:

A 3 $$p_{k+1}={\hat{p}}_{k}+\Delta \beta_{k}$$

and

A 4 $$m_{k+1}=M({\hat{p}}_{k+1})+\gamma_{k+1},$$

where (⋅)_*k*_:=(⋅)_*τ*_*k*__, Δ***β***_*k*_=***β***_*k*+1_−***β***_*k*_ and ${\hat{p}}_{k}$ denotes the updated parameter. Within the Monte-Carlo set-up that we adopt here, let ${\left\{{\hat{p}}_{0}(j)\right\}}_{j=1}^{n_{E}}$ denote the initial set of parameter realizations drawn from a prior probability distribution. Here, *n*_*E*_ denotes the ensemble size. The particle-wise prediction equation that propagates the updated ensemble ${\left\{{\hat{p}}_{k}(j)\right\}}_{j=1}^{n_{E}}$ at *τ*_*k*_ to generate the predicted ensemble ${\left\{p_{k+1}(j)\right\}}_{j=1}^{n_{E}}$ for the (*k*+1)th iteration follows from (A 3) as

A 5 $$p_{k+1}(j)={\hat{p}}_{k}(j)+\Delta \beta_{k}(j),\;j=1,\ldots ,n_{E}.$$

The update step, aimed at driving the measurement-prediction misfit to a zero-mean noise, would essentially involve solving the Kushner–Stratonovich (KS) equation [59,60] well known in nonlinear filtering theory. Unfortunately, the KS equation offers a closed-form analytical formula to the posterior parameter distribution only for the case of linear Gaussian measurements. In such a case, the KS equation is known to reduce to a Kalman–Bucy filter update [61]. Here, we follow a nonlinear ensemble version of the Kalman filter [62] which would indeed have reduced to a Kalman-like update had the measurement operator M been linear (see below). Specifically, the particle-wise update equation is given by

A 6 $${\hat{p}}_{k+1}(j)=p_{k+1}(j)+G_{k+1}(m_{k+1}-M(p_{k+1}(j))),\;j=1,\ldots ,n_{E}.$$

Here, $G\in R^{n_{P}\times n_{M}}$ is called a gain matrix, bearing resemblance with the Kalman gain and is given by $G_{k+1}=P_{k+1}M_{k+1}^{T}{\left(M_{k+1}M_{k+1}^{T}+\Sigma_{\gamma }\Sigma_{\gamma }^{T}\right)}^{-1}$ [63]. $P_{k+1}\in R^{n_{P}\times n_{E}}$ and $M_{k+1}\in R^{n_{M}\times n_{E}}$ denote the perturbation matrices corresponding to the parameter and measurement ensembles, respectively. The perturbation matrices may be looked upon as the square roots of ensemble covariance matrices and are evaluated as follows:

A 7 $$P_{k+1}=\frac{1}{\sqrt{n_{E}-1}}\left[p_{k+1}(1)-\frac{1}{n_{E}}\sum_{j=1}^{n_{E}}p_{k+1}(j),\ldots ,p_{k+1}(n_{E})-\frac{1}{n_{E}}\sum_{j=1}^{n_{E}}p_{k+1}(j)\right]$$

and

A 8 $$\begin{array}{r} M_{k+1}=\frac{1}{\sqrt{n_{E}-1}}\left[M(p_{k+1}(1))-\frac{1}{n_{E}}\sum_{j=1}^{n_{E}}M(p_{k+1}(j)),\ldots ,M(p_{k+1}(n_{E}))-\frac{1}{n_{E}}\sum_{j=1}^{n_{E}}M(p_{k+1}(j))\right]. \end{array}$$

Essentially, once the prediction ensemble ${\left\{p_{k+1}(j)\right\}}_{j=1}^{n_{E}}$ is generated, each of these realizations is input to the forward model to generate the predicted/computed measurements, ${\left\{M(p_{k+1}(j))\right\}}_{j=1}^{n_{E}}$. Each predicted realization is then given an ‘additive push’ that is proportional to the magnitude of its corresponding measurement-prediction misfit. Ideally, when ${\left\{{\hat{p}}_{k+1}(j)\right\}}_{j=1}^{n_{E}}$ converges to the optimal solution, the misfit ${\left\{m_{k+1}-M({\hat{p}}_{k+1}(j))\right\}}_{j=1}^{n_{E}}=0$ modulo small, zero-mean fluctuations determined by ***γ***_*k*+1_.

A term-by-term comparison of (A 6) with a regularized Gauss–Newton (GN) update [42] reveals an interesting feature: the gain matrix ***G***_*k*+1_ apparently plays the role of a Fréchet derivative and may thus be considered as the directional component of COMBEO. Nevertheless, the gain term is free of the gradient calculations as in a generalized Newton method. To understand how (A 6) reduces to a Kalman-like update for the linear case, let us assume that M represents the linear measurement operator. In that case, one could use the formula for the Kalman gain, which is $G_{k+1}^{\mathrm{Kal}}=P_{k+1}M_{k+1}^{T}{\left(M_{k+1}P_{k+1}M_{k+1}^{T}+\Sigma_{\gamma }\Sigma_{\gamma }^{T}\right)}^{-1}$, where ***P***_*k*+1_ is the process error covariance matrix. Rewriting $P_{k+1}=P_{k+1}P_{k+1}^{T}$ in the above expression for the Kalman gain and noting that $M_{k+1}P_{k+1}=M_{k+1}$ for the linear case (A 8), we arrive at our original expression for ***G***_*k*+1_ in (A 6).

The parameter estimate evaluated at each iteration is the empirical mean given by

A 9 $${\bar{\hat{p}}}_{k+1,n_{E}}=\frac{1}{n_{E}}\sum_{j=1}^{n_{E}}{\hat{p}}_{k+1}(j).$$
